# Prevalence and Risk Factors of Headache Associated with COVID-19

**DOI:** 10.3390/jcm13175013

**Published:** 2024-08-24

**Authors:** Oľga Duraníková, Simona Horváthová, Peter Sabaka, Michal Minár, Veronika Boleková, Igor Straka, Peter Valkovič

**Affiliations:** 1 2nd Department of Neurology, Faculty of Medicine, Comenius University Bratislava, 813 72 Bratislava, Slovakia; olga.duranikova@gmail.com (O.D.); simonah09@gmail.com (S.H.); mmminar@gmail.com (M.M.); veronika.bolekova@gmail.com (V.B.); 2 Department of Infectology and Geographical Medicine, Faculty of Medicine, Comenius University Bratislava, 813 72 Bratislava, Slovakia; petersabaka@gmail.com; 3 Faculty of Psychology, Institute of Clinical Psychology, Pan-European University, 821 02 Bratislava, Slovakia; 4 Institute of Normal and Pathological Physiology, Centre of Experimental Medicine, Slovak Academy of Sciences, 813 71 Bratislava, Slovakia

**Keywords:** COVID-19, headache, post-COVID syndrome, biomarkers

## Abstract

**Background:** Headache is a prevalent and disabling non-respiratory symptom of COVID-19, posing a persistent challenge in post-COVID syndrome. This study aimed to determine the prevalence, phenotypes, risk factors and biomarkers associated with COVID-related headaches. **Methods:** A retrospective analysis of 634 hospitalized COVID-19 patients was conducted, with 295 participants being followed up 12–15 months post-discharge via telephone call. Initial laboratory workups, including complete blood count and various biochemical parameters, were compared between headache and non-headache groups. **Results:** One-third of hospitalized patients experienced headaches, predominantly younger individuals (*p* < 0.001) and women (*p* = 0.002). Non-dominant headaches were characterized as dull (56.9%) and holocranial (26.5%), while dominant headaches were unilateral (31.3%) with photophobia (34.3%) and nausea (56.3%). Persistent headaches were unilateral (40%) and pulsating (38%) with phonophobia (74%). Decreased CD4 T cells independently predicted COVID-associated headaches, with elevated IL-6 levels noted in the dominant-headache group (*p* = 0.040). Remarkably, 50% of patients reported persistent headaches 12–15 months post-infection. Dexamethasone administration significantly reduced the likelihood of long-COVID headaches (52% vs. 73%, *p* = 0.029). **Conclusions:** Headache was present in one-third of patients with heterogenous phenotypes: tension headache in the non-dominant group, and migraine in the dominant and persistent headache groups. Persistent headache remains a challenge, with dexamethasone showing potential in reducing its incidence, emphasizing the need for tailored approaches in managing long-COVID headaches.

## 1. Introduction

Coronavirus disease (COVID-19) is the clinical presentation of an infection caused by severe acute respiratory syndrome coronavirus 2 (SARS-CoV-2), which became a global pandemic in 2020.

Although respiratory symptoms dominate in the clinical course of COVID-19 in a great majority of patients, various neurological presentations and associated complications have been described.

Since the first reports of COVID-19, headache has been considered one of the most common and disabling neurological symptoms, affecting 6–70% of the infected population [1]. The broad range of headache frequency is mainly due to methodological differences between studies (collection of data via interview vs. medical records), patients’ population (outpatient vs. inpatient), study design (retrospective vs. prospective), severity of the disease and also the main focus of the study (headache vs. general COVID-19 survey).

Exact pathophysiological mechanisms are still not clearly established, although several hypotheses have been proposed. In one hypothesis, coronavirus enters the host cells through binding to angiotensin-converting enzyme 2 (ACE2) receptors, which are also responsible for the regulation of immune response. This process leads to a dysregulated immune response involving the innate immune system, NOD-like Receptor Protein 3 (NLRP3) inflammasomes and proinflammatory cytokines such as interleukin 6 (IL-6) that induce local and remote effects on vessels and tissues. Another hypothesis involves direct invasion of the trigeminal nervous system via the nasal cavity, although evidence on neurotropism of SARS-CoV-2 still remains controversial [2]. In clinical practice, headache associated with COVID-19 has also become one of the most persistent and difficult-to-treat symptoms in the setting of post-COVID syndrome. In our study, our primary goal was to assess the prevalence and characteristics of headache in hospitalized patients with COVID-19. Our second goal was to search for possible risk factors and biomarkers attributed to different phenotypes of COVID-19-associated headache.

## 2. Materials and Methods

### 2.1. Study Population and Data Collection

We conducted a retrospective study which included hospitalized patients with COVID-19 infection, confirmed by polymerase chain reaction for SARS-CoV-2-RNA on nasopharyngeal swabs at hospital admission between March 2020 and April 2021. Informed consent was obtained on admission to the hospital (written form) in accordance with the Declaration of Helsinki, and the local ethics committee (Academic Derer’s University Hospital, Bratislava) approved study protocol No. 02/2020. Verbal informed consent was obtained during the telephone survey according to protocol No. 18/2023.

We included 634 patients who were hospitalized at the Department of Infectology and Geographical Medicine of the Comenius University Faculty of Medicine and University Hospital Bratislava, Slovakia. For further analysis, we excluded patients who died (n = 67), patients who were older than 75 years (n = 95) and patients who did not want to be part of the survey or from whom telephone contact details were not obtained during their hospital stay (n = 214).

We collected data from the remaining 295 participants using the hospital information system, which contained patients’ demographic data, complete personal history and laboratory workups. Afterwards, these patients were contacted by telephone by neurologists with expertise in headache disorders 12–15 months after their hospital admission due to COVID-19 in order to obtain the following data. The first part of the telephone survey focused on the presence of headache during COVID-19 infection, which divided our patients into headache and non-headache groups. Patients in the headache group were consequently asked about the character of the headache, localization, intensity, dominancy and response to analgesics, and the impact of headache on their everyday lives. At the same time, we correlated data regarding headache characteristics in the admission hospital reports.

Features of headache in the headache group were further classified into 3 categories:

- **Time:** patients with persisting and non-persisting COVID-related headache
- **Dominancy:** patients with headache as a dominant and non-dominant symptom (reported by patients)
- **History:** patients with or without preexisting diagnosis of primary headache

**Figure 1:**
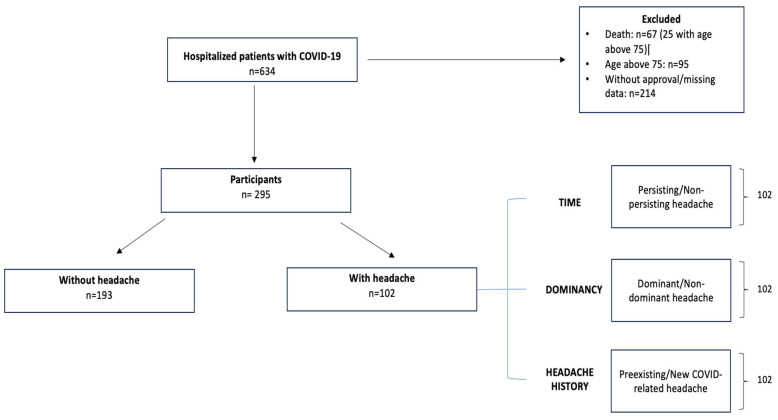
Study sample.

For both the headache and non-headache groups, we compared initial laboratory workups at admission to the hospital, including complete blood count, biochemical, coagulation and immunological parameters. The complete flowchart is shown in Figure 1.

### 2.2. Statistical Analysis

The data were analyzed using the Statistical Package for Social Sciences (SPSS, Statistics 26.0, IBM Corp, Armonk, NY, USA). Demographic and clinical parameters were analyzed using descriptive statistics. For comparative analysis, we used the Chi-Square (categorical variables), T-test and Mann–Whitney test (quantitative variables with normal and non-normal distribution). We used multivariate binary logistic regression (method “enter”) to confirm the association of headache with other variables, where we included predictors that were statistically significant in comparative analysis. *p* value ≤ 0.05 was considered to be significant.

## 3. Results

### 3.1. Headache Prevalence and Characteristics

From the set of included subjects with COVID-19, we identified 34.6% (n = 102; headache group) of participants suffering from new headaches and/or worsening of preexisting headache in relation to COVID-19.

In the headache group, the most common quality of headache was dull in 56.9% (n = 58). The most common localization of headache was holocranial, in 26.5% (n = 27), followed by hemicranial headache, in 25.5% (n = 26). Nausea was the most common accompanying symptom in 43.1% (n = 44) of patients, followed by photophobia in 21.6% (n = 22), phonophobia in 14.7% (n = 15) and vomitus in 7.8% (n = 8) of cases. In 36.3% (n = 37), headache did not respond to analgesics.

Headache resolved in parallel with the resolution of other symptoms of acute infection in 34.3% (n = 35) of subjects, within 2 consecutive weeks in 11.8% (n = 12), from 2 to 4 weeks in 12.7% (n = 13) and remained persistent until the moment of survey (12–15 months after their hospital admission) in 41.2% (n = 42) of patients.

Table 1 provides specific information on the characteristics of headache.

Comparing both groups, there were significantly more women in the headache group compared to the non-headache group (65.4% vs. 34.6%, *p* = 0.002). Patients with COVID-related headache were significantly younger (*p* < 0.001), and they spent fewer days at the hospital (*p* = 0.005). Patients with headache had significantly lower CD4+ T cells (*p* = 0.038) and lower serum levels of fasting glucose (*p* = 0.008). There was no significant difference in the levels of CRP, procalcitonin, vitamin D, CD8+T cells, fibrinogen and D-dimer. All data are shown in Table 2.

Using multivariate logistic regression analysis, we confirmed lower CD4+ T-lymphocytes as an independent predictor of COVID-19-related headache (*p* = 0.011). Another independent predictor was younger age (*p* = 0.005). Complete data are shown in Table 3.

Comparing both groups, we did not find a significant association with administered anti-COVID drugs or patients’ comorbidities. Complete data are shown in Table 4 and Table 5.

### 3.2. Dominant vs. Non-Dominant Headache

Headache was considered the dominant and most bothersome symptom in 31.4% (n = 32) of patients with headache.

Hemicranial headache was present in 31.3% of the dominant group in comparison with 12.9% in the non-dominant group. Pulsating quality was present in 25% versus 11.4%. It was accompanied by photophobia in 34.4% versus 15.7%, with nausea in 56.3% versus 37.1% and vomitus in 18.3% versus 2.9%.

**Figure 2:**
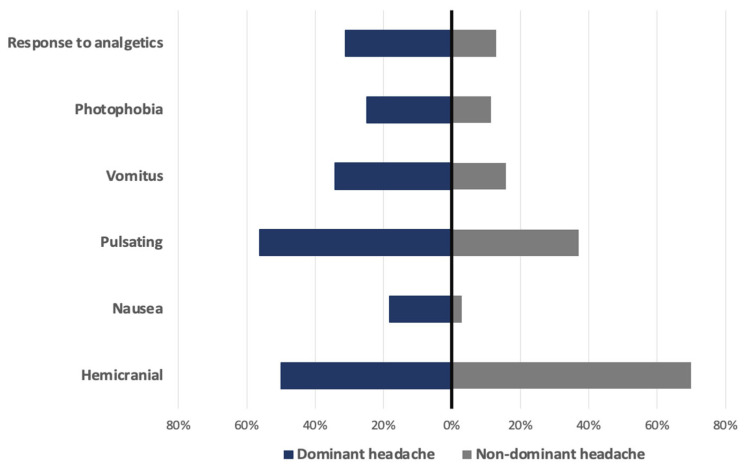
Comparison between main characteristics in dominant and non-dominant headache.

**Figure 3:**
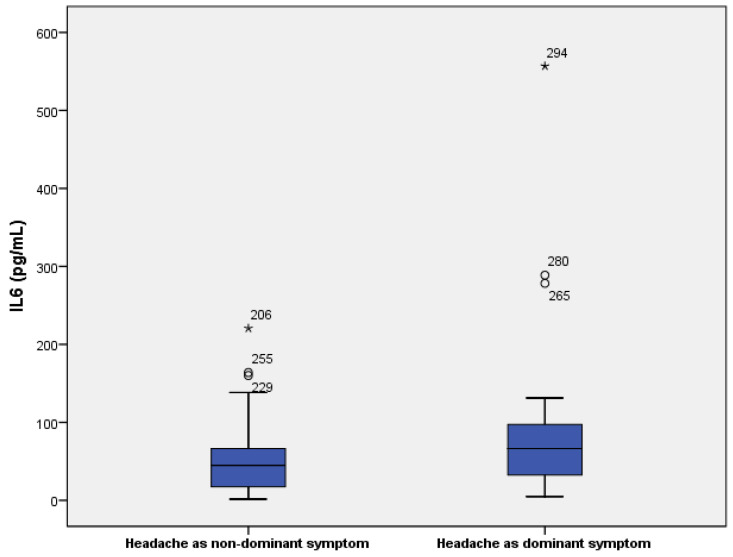
Serum levels of IL-6 in patients with headache as a dominant and non-dominant symptom. ○ are mild outliers and * are extreme outliers.

**Figure 4:**
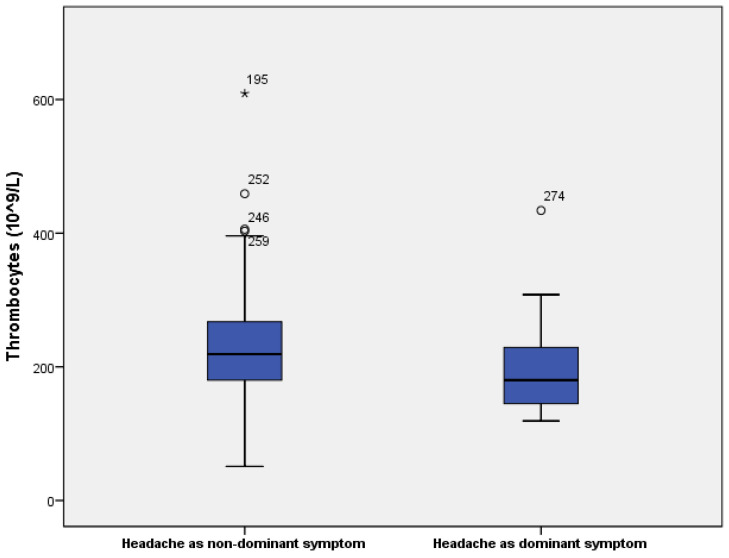
Thrombocytes in patients with headache as a dominant and non-dominant symptom. ○ are mild outliers and * are extreme outliers.

In 50% of patients in the dominant symptom group, the headache was responsive to analgesics, compared to 70% responsiveness in the group with headache as a non-dominant symptom. Patients with headache as a dominant symptom also had significantly higher serum levels of IL-6 (66.5 vs. 44.8 pg/L, *p* = 0.04) and lower count of thrombocytes (180 vs.219 × 10^9^/L, *p* = 0.016). All data are shown in Table 6 and Table 7 and Figure 2, Figure 3 and Figure 4.

Using a multivariate logistic regression model (method enter) we confirmed that lower age predicted headache as a dominant symptom (*p* = 0.011). All data are shown in Table 8.

### 3.3. Persisting vs. Non-Persisting Headache

**Figure 5:**
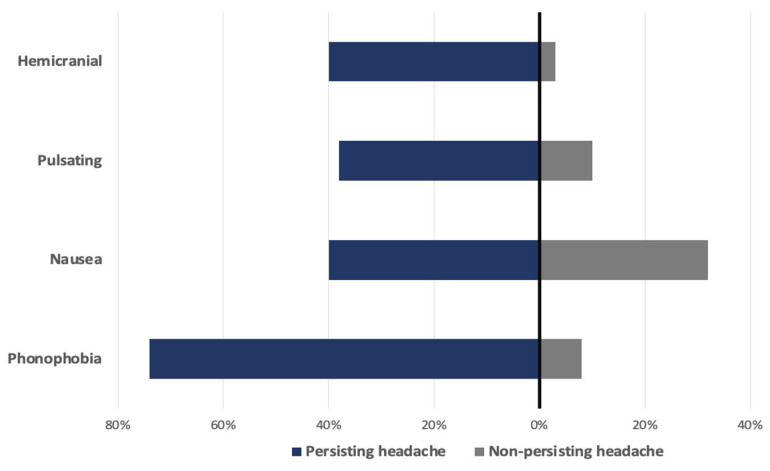
Comparison between main characteristics of persisting and non-persisting headache.

Persisting headache for more than 15 months was present in 41.2% (n = 42) of subjects from the headache group. The most common quality was pulsating, in 38%, and it was unilateral in 40%. It was accompanied by nausea in 60% and phonophobia in 74% of patients. Whereas no specific laboratory marker or comorbidity was associated with persisting headache, we found a significant association between persisting headache and treatment with dexamethasone. Patients treated with dexamethasone during hospitalization had a lower chance of developing persisting headache (52% vs. 73%, *p* = 0.029). All data are shown in Table 9, Table 10, Table 11 and Table 12 and Figure 5.

Using multivariate logistic regression analysis (Enter method), we confirmed that the use of dexamethasone was a negative independent predictor of persisting headache (OR = 0.4, *p* = 0.031). Data are shown in Table 13.

### 3.4. Preexisting Primary Headache vs. No Preexisting Primary Headache

Preexisting primary headache was reported in 14 patients (13.8%), with migraine accounting for 9.8% (n = 10) and tension-type headache for only 4% (n = 4) of patients. Other primary headaches were not present. Sixty percent of patients with migraine reported increased intensity and frequency of their previous headache, and the same percentage of patients were less responsive to their previous migraine medication. A change in pattern to tension-type headache was identified in two patients (20%). Patients with tension-type headache reported both higher intensity and frequency of headache in one case (25%). In 50% of patients, headache did not respond to previous medication. A change in migraine pattern to tension-type-like headache was observed in one case (25%).

## 4. Discussion

### 4.1. Risk of Headache

This study proved that headache is a very common symptom of COVID-19, affecting more than one-third of patients (34.6%). Other comparable studies of hospitalized patients referred to the prevalence of COVID-19 headache in 27.9% and 28.9%, respectively [3]. Another epidemiological study using retrospective design referred to a markedly lower prevalence of 13% [4].

Although headache is a common symptom of various viral infections, defined as ‘Headache attributed to systemic viral infection’ (9.2.2 according to ICHD-3), species-specific epidemiological data are limited. Headache was associated with infection of the ZIKA virus during the Zika–Dengue epidemics in Cuba, where headache prevalence reached 50.8% [5]. In the same year, Sampaio Rocha Filho et al., conducted a study focusing on headache in HIV patients, with a prevalence of 87% [6]. Interestingly, despite the diagnosis of new daily persistent headache (NDPH) that was first mentioned in the literature in 1980, some data suggest that headache was one of the primary manifestations of the 1890 viral pandemics, also known as the Russian or Asiatic flu. It seems that headache was present in 75–83% of patients, with some reports proposing persisting headache for months or years, although the true proportion of headache that developed into true ICHD-defined NDPH is not known [7].

According to available data, patients with COVID-19-associated headache are usually younger, and headache itself is associated with a lower rate of mortality [8,9,10]. Patients in our sample were younger and spent fewer days in the hospital. It was not possible to establish the association of headache with mortality rate, as patients with the most severe course of COVID-19 died and were not included.

In addition to younger age, lower CD4+ T cells count was associated with a higher risk of COVID-related headache. However, we acknowledge that other potential confounding factors, such as the use of other medications (e.g., analgesics, corticosteroids, antivirals) and preexisting neurological conditions (e.g., history of migraines, tension-type headaches or other neurological disorders) may have also influenced these outcomes. CD4+ T cells play a key role in the coordination of the immune system response to acute and chronic viral infection by activating multiple cells of the innate immune system, B cells, CD8+ T cells and non-immune cells. They are also necessary for the establishment of long-term cellular and humoral antigen-specific immunity, which is pivotal for life-long protection from many viral infections [11]. In terms of COVID-19 infection, CD4+ T cells also have an important role in adaptive immunity against SARS-CoV-2 virus. Moreover, specific CD4+ T cell response is positively associated with long-term persistence of neutralizing antibodies [7]. Furthermore, vector and mRNA vaccines induce specific CD4+ T cell responses, which may also be involved in the effectiveness of these vaccines [11]. Additionally, decreased CD4+ T cell response against the spike protein was associated with increased severity of neurological symptoms, which may suggest an important role of T cell response in preventing a severe course of neurological long COVID. Interestingly, Leng et al., documented that COVID-19 vaccination with mRNA vaccines increased T cell response and subsequently helped reduce the severity of neurological symptoms in long COVID [12].

From the very beginning of the pandemic, the resemblance of COVID headache to headache attributed to systemic viral infection, as well as often-accompanying symptoms (myalgia, arthralgia or lightheadedness), were indicative that innate immune response and cytokine storm may play an important role in the process. Various studies have, therefore, focused on identifying the cytokine profile of patients with headache attributed to COVID-19 infection that may also pose as therapeutic targets [3].

Interleukin-6 (IL-6), an important proinflammatory cytokine, has been proven to play an important role in the neurological manifestation of COVID-19. Moreover, IL-6 was also identified as a potential biomarker of severe COVID-19 infection. With regard to the pathophysiological mechanism of IL-6 and migraine, IL-6 is considered to be one of the triggers of the trigeminovascular system [13]. Preclinical studies in rat models also showed that IL-6 in heat conditions was associated with the release of calcitonin-gene peptide (CGRP), both of which are processes that are pivotal in the pathophysiology of migraine attack [14]. In our cohort, we did not find a correlation between IL-6 and headache as a non-dominant symptom; however, higher levels of IL-6 were associated with headache as the dominant symptom (*p* = 0.04). The difference may suggest that a more severe cytokine storm during infection could be a potential factor. To our knowledge, this is the first study with a sub-selection of patients with headache as the dominant symptom. Therefore, further studies are needed.

### 4.2. Types of Headache

Despite the high prevalence and associated disability of COVID-19-related headache, it seems its phenotype is ambiguous and is related to the disease state and certain symptoms (mostly fever) [14]. Nevertheless, several studies have described two main phenotypes of COVID-19-associated headache. The first one is a headache with tension-type features with a pressing quality of headache, mild or moderate intensity and not aggravated by physical activity. The second most common phenotype has migraine-like features with a pulsating quality, associated with nausea and related to high intensity and disability.

Because of this heterogeneity, we separately analyzed the following subgroups: (a) headache as a dominant and non-dominant symptom, (b) patients with persisting headache and (c) patients with preexisting primary headache.

Headache is not only one of the most common but also one of the most disabling neurological symptoms of COVID-19. Various studies reported a significant negative impact of headache on quality of life and deterioration of social and psychological activities [15]. For that reason, we also decided to divide patients into dominant and non-dominant groups in accordance with headache being considered the most dominant and bothersome symptom for the patients, although it is not an official classification according to ICHD-3.

Headache as a non-dominant symptom mostly resembled tension-type headache, which corresponds to the 9.2.2. definition of headache attributed to systemic viral infection according to ICHD-3 [8,16].

On the other hand, headache as a dominant symptom had predominantly migraine-like features. It was unilateral in 31.3% of cases, had a pulsating quality in 25% of patients and was associated with nausea and photophobia in 56.3% and 34.4%, respectively. In comparison with the study that was published by Garcia-Azorín et al., headache was considered the dominant and most bothersome symptom in 14.3% of hospitalized patients and 21% of outpatients, although studies focusing specifically on this subgroup are limited [17]. Similarly, our research showed that the majority of patients with persisting headache for 12–15 months after the acute infection presented with migraine-like features.

#### 4.2.1. Persisting Headache

It is well established that, in many patients, various symptoms persist for weeks or even months after the acute infection. This period, also known as long-COVID syndrome, can be present in patients with a previous COVID-19 diagnosis, with presentation of symptoms at least 4 weeks and not later than 2 months after the acute phase of the infection and that are present for at least 3 months [18]. Headache is considered to be one of the most common neurological symptoms in the long-COVID phase, along with memory impairment, poor concentration, depression and sleep disorders [19,20,21]. In our cohort, we confirmed 41.2% of patients with persistent long-COVID headache after 12–15 months, which may be associated with the fact that all of our patients required hospitalization and had other comorbidities such as arterial hypertension or diabetes. In comparison, in the retrospective Spanish study published by Aparisi et al., 14.3% of patients had long-COVID headache with a mean total duration of 9 months until complete resolution [22]. The results of another study, conducted in an outpatient long-COVID clinic in Japan, revealed a prevalence of persistent headache in 23.4% of outpatients [19].

Despite its frequent occurrence and the associated disability, our knowledge about long-COVID headache is still limited, and exact characteristics, pathophysiology and efficient treatment are still not fully understood. There are, however, several hypotheses on its underlying pathophysiology. The first one proposes persistent immune system activation with bio-humoral response supported by modified levels of cytokines and interleukins observed in some studies. Also, according to research conducted by Swank et al., that focused on possible biomarkers associated with post-acute sequelae of coronavirus disease 2019 (PASC), the coronavirus 2 spike protein was detected in PACS patients up to 12 months after diagnosis, which may suggest that a reservoir of active virus persists in the body [23]. Moreover, based on autopsies studies, persistent SARS-CoV-2 RNA was found in multiple anatomic regions, including the brain, up to 230 days after the onset of infection [24]. The second probable hypothesis includes trigeminal system activation in patients with a genetic predisposition to migraine or in patients with preexisting headache. In the study that was published by Garcia-Azorin et al., persistent headache at 9 months was more common in patients with migraine features at baseline; however, further studies are needed [17]. The third proposed mechanism involves possible structural and functional brain changes, as suggested by research published by Planchuelo-Gómez et al. The results of this longitudinal project revealed increased cortical surface area and grey matter volume in patients with long-COVID headache with predominancy in the primary olfactory cortex. Moreover, white matter changes have been observed in COVID-19 survivors with higher axial diffusivity in corona radiata and internal and external capsules, indicating some degree of white matter axonal alterations, which may be related to the persistence of headache, but the specificity of these findings in association with headache is still to be confirmed [25].

Long-COVID headache may present in a form similar to new daily persistent headache (NDPH) because of its predominant temporal relationship and poor response to treatment. Nevertheless, not all headaches present on a daily basis. This may reflect disease severity and the use of analgesics; therefore, in some cases, intermittent or chronic daily headache may occur. It also seems that long-COVID headache does not have a uniform phenotype and may present in the form of migraine or tension-type headache. In our study, the majority of patients had headache with migraine features, presenting as pulsating headache in 38%, unilateral in 40% and associated with nausea and phonophobia in 60% and 74%, respectively.

While no specific laboratory marker was associated with persisting headache, we found that patients who were treated with dexamethasone during hospitalization had a lower chance of developing persisting headache. Similar results were mentioned in an extensive study published by Grundmann et al., where treatment with dexamethasone and remdesivir was associated with a lower risk of developing neurological complications associated with COVID-19 infection [26].

#### 4.2.2. Preexisting Primary Headache

In our group, the majority of patients experienced de-novo COVID-19-associated headache, while only 13.8% of patients had a preexisting diagnosis of primary headache, with migraine accounting for 9.8% and tension-type headache for only 4% of patients.

Although data on preexisting primary headaches and COVID-19 are limited, it appears that the association between migraine and COVID-19 is the most researched one. It seems that the prevalence of COVID-19 infection does not differ in patients with migraine in comparison with the general population; however, patients with preexisting migraine are more prone to develop headache in the acute phase, with earlier and more severe manifestations with prolonged duration [14]. In 60% of our patients with preexisting migraine, there was an increase in headache intensity and frequency. In 30% of cases, headache changed its typical localization. In two-thirds of patients, migraine was not responsive to previous medication. Our results are similar to the research conducted by Al Hashel et al., that focused on preexisting primary headaches with concomitant COVID-19 infection. Their study revealed that a higher frequency of migraine was present in 59.3% of patients and that 63.4% experienced a more severe course of migraine attack [27]. Migraine attack, in general, is also linked to hormonal changes and, thus, phases of the menstrual cycle, with especially menstruation being the most common trigger for migraine. Interestingly, according to a study published by Pelayo-González et al., women in the luteal phase of the cycle had a distinct electroencephalographic pattern in comparison with healthy controls [28]. While electrophysiological techniques are used to study neural activity and have been used in headache research, this has not been explored in patients with COVID-19-related headache and further studies are needed.

In our group of patients with preexisting tension-type headache, we documented increased intensity and frequency of headache in 25% and 50% of cases, respectively. Half of these patients were also less responsive to their previous chronic medication. Twenty-five percent of patients with preexisting tension-type headache experienced a change of pattern from their usual headache to migraine.

### 4.3. Limitations

Our study has several limitations. Firstly, it was a retrospective study, and data were obtained only via the phone; therefore, neurological examination was not performed. Also, all patients included in our study required hospitalization due to COVID-19 infection, which could mask the real proportion of COVID-associated headache, as the majority of patients with headache have a milder course of infection and are therefore treated as outpatients. Additionally, we excluded patients who died with the most serious comorbidities; however, it was necessary due to the retrospective design of our study.

Another limitation of our study is based on the fact that laboratory tests were obtained on the first day of hospital admission, which does not always represent the start of patients’ symptoms. For that reason, we also did not proceed with follow-up biomarkers, as the time frame was individual for each patient and was dependent on the patient’s clinical status. Also, we mostly tried to identify the possible laboratory biomarker at the beginning of the infection, that could predict the possibility of persisting headache.

The absence of a control group can be a limitation; however, we tried to minimize the risk by specifically asking about preexisting primary headache. Furthermore, we deliberately asked questions related to COVID-19 infection.

Another limitation of our study is that we did not correlate headache with the menstrual periods of our female patients because of the retrospective design of our study as well as the relatively long time period since the COVID-19 infection.

## 5. Conclusions

We confirmed headache as a common symptom of COVID-19 infection in more than one-third of hospitalized patients, with predominance in younger people and women. Patients with headache had a significantly shorter length of hospitalization, which supports hypotheses of a milder course of infection.

We showed that COVID-19-associated headache is not uniform and depends on various factors. The tension-type-like headache phenotype was most prevalent in patients with headache as a non-dominant symptom, while migraine features prevailed in patients with dominant and persisting headache. We confirmed persisting headache for 12–15 months after acute infection in almost 50% of our patients, which reflects the ongoing challenge of long-COVID headache management in everyday clinical practice. The heterogeneity of COVID-19-associated headache was also reflected in identifying associated biomarkers. Decreased CD4+ T cells were found as an independent predictor of COVID-associated headache. We found elevated levels of IL-6 in the subpopulation of patients with headache as a leading symptom, which may indicate a more severe cytokine storm as an underlying mechanism. We documented a remarkable proportion of patients with persisting headache 12–15 months after acute onset of COVID-19. Interestingly, patients with administered dexamethasone had a statistically lower probability of developing long-COVID headache. This has relevant implications for clinical practice, as long-COVID headache remains a therapeutic challenge worldwide, and specific recommendations and a tailored approach are needed.

## Figures and Tables

**Table 1:** Characteristics of patients with headache.

	%	n
**Character of headache**		
Dull	56.90	58
Pulsating	21.60	22
Stabbing	18.60	19
Other	2.90	13
**Localization of headache**		
Holocranial	26.50	27
Hemicranial	25.50	26
Occipital and neck region	19.60	20
Temporal	18.60	19
Retrobulbar	1.00	1
Frontal	25.50	26
Accompanying symptoms *		
Nausea	43.10	44
Vomitus	7.80	8
Photophobia	21.60	22
Phonophobia	14.70	15
Worsening with physical activity	7.40	13
**Response to analgesics**		
Yes	63.70	65
No	36.30	37
**Duration of headache**		
With resolution of other symptoms	34.30	35
Up to 2 weeks	11.80	12
15–30 days	12.70	13
Persisting for 12–15 months	41.20	42
**Preexisting primary headaches**		
Migraine	9.80	10
Tension-type headache	4.00	4
Other primary headaches	0.00	0
No primary headache	86.20	88
**Limitations in daily activities**		
Yes—markedly	25.50	30
No	29.40	26
Yes—mildly to moderately	45.10	46
**Hospitalization at the ICU**		
Yes	13.90	12
No	86.10	
**Oxygen therapy**		
No oxygen therapy	19.80	20
Low-flow oxygen therapy	69.30	70
HFNO	9.90	10
Mechanical ventilation	1	1
**Dexamethasone**		
Yes	64.71	66
No	35.29	36

ICU—intensive care unit. * Patient could have referred zero to several accompanying symptoms.

**Table 2:** Clinical and laboratory characteristics of patients with and without headache.

	All Participants	Headache Group	Non-Headache Group	*p*
**Age** (years)	58.00 (47.00; 66.00)	51.00 (40.75; 61.25)	61.00 (51.50; 68.00)	**<0.001**
**Hospitalization length** (days)	9.00 (6.00; 13.00)	8.00 (5.00; 11.25)	9.00 (7.00; 14.00)	**0.005**
**CRP** (mg/L)	85.29 (30.77; 138.93)	83.03 (35.38; 145.25)	87.06 (25.53; 138.93)	0.988
**IL-6** (pg/L)	49.70 (20.72; 80.56)	48.30 (21.40; 76.30)	50.70 (20.50; 82.10)	0.681
**Procalcitonin** (ng/mL)	0.07 (0.04; 0.13)	0.07 (0.04; 0.13)	0.08 (0.04; 0.14)	0.295
**Vitamin D** (nmol/L)	28.90 (19.80; 38.25)	32.40 (21.25; 38.10)	27.60 (18.85; 38.55)	0.204
**Thrombocytes** (× 10^9^/L)	207.00 (166.00; 261.75)	206.10 (161.50; 256.25)	208.50 (166.75; 273.00)	0.545
**CD4+ T cells** (abs)	0.32 (0.25; 0.57)	0.30 (0.25; 0.42)	0.43 (0.34; 0.67)	**0.038**
**CD8+ T cells** (abs)	0.20 (0.10; 0.32)	0.18 (0.11; 0.31)	0.23 (0.10; 0.34)	0.406
**Fibrinogen** (g/L)	5.10 (4.20; 6.00)	5.10 (4.20; 5.90)	5.05 (4.28; 6.20)	0.542
**D-dimer** (mg/L)	0.77 (0.47; 1.20)	0.70 (0.47; 1.08)	0.81 (0.51; 1.31)	0.081
**Fasting glucose** (mmol/L)	6.60 (5.80; 8.00)	6.20 (5.60; 7.40)	6.80 (5.90; 8.80)	**0.008**

Numerical variables were expressed as median, interquartile range (IQR). Bold is used to highlight statistical significance, *p* ≤ 0.05.

**Table 3:** Multivariate binary logistic regression model for patients with headache as a non-dominant symptom-Enter analysis.

	B	OR	*p*	95% CI	
**CD4+ T cells** (abs.)	2.920	18.534	**0.011**	1.969	174.445
**Age**	0.082	1.086	**0.005**	1.026	1.150
**Sex**	−1.042	0.353	0.115	0.097	1.289
**Fasting glucose**	0.103	1.109	0.443	0.852	1.444
**Hospitalization length**	0.069	1.071	0.108	0.985	1.166
**constant**	−6.106	0.002	0.001		

(CI—confidence interval, OR—odds ratio). Bold is used to highlight statistical significance, *p* ≤ 0.05.

**Table 4:** Comorbidities in patients with and without headache.

	Headache Group (n)/%	Non-Headache Group (n)/%	*p*
**Diabetes**	13/12.75	33/17.10	0.330
**Heart failure**	1/0.98	8/4.14	0.134
**Myocardial infarction**	3/2.94	11/5.70	0.291
**Dialysis**	0/0	1/0.52	0.467
**Chronic obstructive pulmonary disease**	1/0.98	6/3.12	0.254
**Bronchial asthma**	8/7.84	19/9.84	0.575
**Stroke**	2/1.96	7/3.63	0.431
**Arterial hypertension**	42/41.18	93/48.19	0.252
**Dementia**	0/0	3/1.56	0.206
**Peripheral arterial disease**	0/0	2/1.04	0.303
**Atrial fibrilation**	2/1.96	6/3.11	0.566
**Sepsis at admission**	0/0	1/0.52	0.467

**Table 5:** Administered anti-COVID-19 drugs in patients with and without headache.

	Headache Group (n)/%	Non-Headache Group (n)/%	*p*
**Dexamethasone**	66/64.71	127/65.80	0.851
**Remdesivir**	30/29.41	39/20.21	0.068
**Olumiant**	23/22.55	56/29.02	0.233
**Favilavire**	13/12.75	14/7.25	0.368
**Ivermectin**	4/3.92	5/2.60	0.527
**Colchicin**	1/0.98	3/1.55	0.684

**Table 6:** Characteristics of patients with headache as a dominant symptom.

	Frequency (%)	n
**Character of headache**		
Dull	53.10	17
Pulsating	31.30	10
Stabbing	15.60	5
Other	0.00	0
**Localization of headache**		
Holocranial	25.00	8
Hemicranial	31.30	10
Occipital and neck region	9.40	3
Temporal	21.90	7
**Retrobulbar**		
Frontal	31.30	10
Other	6.30	2
Accompanying symptoms *		
Nausea	56.30	18
Vomitus	18.80	6
Photophobia	34.40	11
Phonophobia	21.90	7
Worsening with physical activity	56.30	18
**Response to analgesics**		
Yes	50.00	16
No	50.00	16
**Duration of headache**		
With resolution of other symptoms	21.9	7
Up to 14 days	9.4	3
15–30 days	15.6	5
Persisting for 12–15 months	53.1	17
**Limitations in daily activities**		
Yes—markedly	21.90	7
No	9.40	3
Yes—mildly to moderately	68.80	22

* Patients could have referred zero to several accompanying symptoms.

**Table 7:** Clinical characteristics of patients with dominant and non-dominant headache.

	Patients with Headache as a Dominant Symptom	Patients with Headache as Non-Dominant Symptom	*p*
**Age** (years)	51.00 (40.75; 61.25)	43.00 (35.00; 56.75)	**0.008**
**Hospitalization length** (days)	8.00 (5.00; 11.25)	9.00 (5.25; 11.75)	0.401
**CRP** (mg/L)	83.03 (35.38; 145.25)	95.00 (29.30; 149.50)	0.949
**IL-6** (pg/L)	48.30 (21.40; 76.30)	66.40 (31.00; 101.40)	**0.040**
**Procalcitonin** (ng/mL)	0.07 (0.04; 0.13)	0.09 (0.05; 0.15)	0.242
**Vitamin D** (nmol/L)	32.40 (21.25; 38.10)	34.10 (24.20; 42.90)	0.290
**Thrombocytes** (× 10^9^/L)	206.10 (161.50; 256.25)	180 (144.00; 233.00)	**0.016**
**CD4+ T cells** (abs)	0.32 (0.25; 0.57)	0.32 (0.25; 0.59)	0.732
**CD8+T cells** (abs)	0.18 (0.11; 0.31)	0.23 (0.13; 0.41)	0.235
**Fibrinogen** (g/L)	5.10 (4.20; 5.90)	4.60 (3.90; 5.90)	0.155
**D-dimer** (mg/L)	0.70 (0.47; 1.08)	0.75 (0.49; 1.08)	0.788
**Fasting glucose** (mmol/L)	6.20 (5.60; 7.40)	5.80 (5.10; 6.85)	**0.029**

Numerical variables were expressed as median, interquartile range (IQR). Bold is used to highlight statistical significance, *p* ≤ 0.05.

**Table 8:** Multivariate binary logistic regression model for dominant and non-dominant headache Enter analysis.

	B	OR	*p*	95% CI	
**Age**	−0.077	0.925	**0.011**	−0.137	−0.018
**IL-6**	0.007	1.007	0.180	−0.003	0.017
**Fasting glucose**	−0.136	0.873	0.560	−0.592	0.320
**Platelets count**	−0.007	0.993	0.141	−0.016	0.002
**Diabetes**	2.587	1.777	0.075	−0.266	5.439
**Constant**	4.355	77.868	0.076		

CI—confidence interval, OR—odds ratio.

**Table 9:** Characteristics of patients with persisting headache.

	%	n
**Character of headache**		
Dull	47.60	20
Pulsating	38.10	16
Stabbing	9.50	4
Other	4.80	2
**Localization of headache**		
Holocranial	28.60	12
Hemicranial	40.50	17
Occipital and neck region	11.90	5
Temporal	26.20	11
Retrobulbar	0.00	0
Frontal	19.00	8
Accompanying symptoms *		
Nausea	59.50	25
Vomitus	7.10	3
Photophobia	26.20	11
Phonophobia	74.00	31
Worsening with physical activity	54.80	23
**Limitations in daily activities**		
Yes—markedly	26.20	11
No	9.50	4
Yes—mildly to moderately	64.30	42

* Patients could have referred zero to several accompanying symptoms.

**Table 10:** Comorbidities in patients with persisting and non-persisting headache.

	Patients with Persisting Headache (n)	Patients with Non-Persisting Headache (n)	*p*
**Diabetes**	6	7	0.735
**Heart failure**	1	0	0.236
**Myocardial infarction**	2	1	0.376
**Chronic obstructive pulmonary disease**	0	1	0.394
**Bronchial asthma**	4	4	0.625
**Stroke**	1	1	0.813
**Arterial hypertension**	14	28	0.139
**Atrial fibrilation**	1	1	0.813
**Hospitalization in ICU**	4	8	0.557

Numerical variables were expressed as numbers. ICU—intensive care unit.

**Table 11:** Administered anti-COVID-19 drugs in patients with persisting headache.

	Yes	No	*p*
**Dexamethasone**	22	20	**0.029**
**Remdesivir**	10	31	0.334
**Olumiant**	9	33	0.821
**Favilavire**	6	35	0.442
**Ivermectin**	0	42	0.088
**Colchicin**	0	41	0.398

Numerical variables were expressed as numbers. Bold is used to highlight statistical significance, *p* ≤ 0.05.

**Table 12:** Clinical characteristics of patients with persisting and non-persisting headache.

	Patients with Headache Persisting for 12–15 Months	Patients with Non-Persisting Headache	*p*
**Age** (years)	53.00 (40.00; 58.00)	47.00 (41.25; 62.00)	0.171
**Hospitalization length** (days)	9.00 (5.00; 10.50)	7.00 (5.25; 11.75)	0.401
**CRP** (mg/L)	88.17 (27.75; 119.00)	76.00 (40.02; 155.08)	0.298
**IL-6** (pg/L)	50.45 (21.80; 78.60)	42.80 (20.73; 73.22)	0.864
**Procalcitonin** (ng/mL)	0.07 (0.04; 0.09)	0.07 (0.04; 0.15)	0.502
**Vitamin D** (nmol/L)	32.25 (22.30; 37.53)	32.45 (19.88; 43.90)	0.744
**Thrombocytes** (× 10^9^/L)	215.00 (161.00; 254.00)	195.00 (165.50; 267.00)	0.305
**CD4+ T cells** (abs)	0.28 (0.27; 0.59)	0.32 (0.14; 0.40)	0.095
**CD8+ T cells** (abs)	0.14 (0.18; 0.31)	0.20 (0.10; 0.26)	0.112
**Fibrinogen** (g/L)	5.10 (3.90; 5.80)	4.60 (4.45; 5.95)	0.120
**D-dimer** (mg/L)	0.70 (0.49; 1.08)	0.77 (0.42; 1.10)	0.389
**Fasting glucose** (mmol/L)	6.20 (5.30; 7.50)	6.20 (5.70; 7.20)	0.552

Numerical variables were expressed as median, interquartile range (IQR).

**Table 13:** Multivariate binary logistic regression model for persisting and non-persisting headache—Enter analysis.

	B	OR	*p*	95% CI	
**Dexamethasone**	0.916	0.400	**0.031**	−1.749	−0.083
**Constant**	0.223	1.250	0.506	−0.043	0.881

CI—confidence interval, OR—odds ratio). Bold is used to highlight statistical significance, *p* ≤ 0.05.

## Data Availability

The original contributions presented in the study are included in the article, further inquiries can be directed to the corresponding author/s.

## References

[1] Caronna E., Pozo-Rosich P. (2021). Headache as a Symptom of COVID-19: Narrative Review of 1-Year Research. Curr. Pain. Headache Rep..

[2] Bauer L., Laksono B.M., De Vrij F.M.S., Kushner S.A., Harschnitz O., Van Riel D. (2022). The Neuroinvasiveness, Neurotropism, and Neurovirulence of SARS-CoV-2. Trends Neurosci..

[3] Trigo J., García-Azorín D., Sierra-Mencía Á., Tamayo-Velasco Á., Martínez-Paz P., Tamayo E., Guerrero A.L., Gonzalo-Benito H. (2021). Cytokine and Interleukin Profile in Patients with Headache and COVID-19: A Pilot, CASE-Control, Study on 104 Patients. J. Headache Pain.

[4] Gonzalez-Martinez A., Fanjul V., Ramos C., Serrano Ballesteros J., Bustamante M., Villa Martí A., Álvarez C., García Del Álamo Y., Vivancos J., Gago-Veiga A.B. (2021). Headache during SARS-CoV-2 Infection as an Early Symptom Associated with a More Benign Course of Disease: A Case–Control Study. Eur. J. Neurol..

[5] Guanche Garcell H., Gutiérrez García F., Ramirez Nodal M., Ruiz Lozano A., Pérez Díaz C.R., González Valdés A., Gonzalez Alvarez L. (2020). Clinical Relevance of Zika Symptoms in the Context of a Zika Dengue Epidemic. J. Infect. Public Health.

[6] Sampaio Rocha-Filho P.A., Torres R.C.S., Ramos Montarroyos U. (2017). HIV and Headache: A Cross-Sectional Study. Headache.

[7] Wang Z., Yang X., Mei X., Zhou Y., Tang Z., Li G., Zhong J., Yu M., Huang M., Su X. (2022). SARS-CoV-2-Specific CD4+ T Cells Are Associated with Long-Term Persistence of Neutralizing Antibodies. Sig Transduct. Target. Ther..

[8] Trigo J., García-Azorín D., Planchuelo-Gómez Á., Martínez-Pías E., Talavera B., Hernández-Pérez I., Valle-Peñacoba G., Simón-Campo P., De Lera M., Chavarría-Miranda A. (2020). Factors Associated with the Presence of Headache in Hospitalized COVID-19 Patients and Impact on Prognosis: A Retrospective Cohort Study. J. Headache Pain.

[9] Gallardo V.J., Shapiro R.E., Caronna E., Pozo-Rosich P. (2022). The Relationship of Headache as a Symptom to COVID-19 Survival: A Systematic Review and Meta-analysis of Survival of 43,169 Inpatients with COVID-19. Headache.

[10] Peng X., Ouyang J., Isnard S., Lin J., Fombuena B., Zhu B., Routy J.-P. (2020). Sharing CD4+ T Cell Loss: When COVID-19 and HIV Collide on Immune System. Front. Immunol..

[11] Rakshit S., Parthiban Ch Mudhavan R., Adiga V., Eveline J.S., Kumar N.C.H., Ahmed A., Shivalingaiah S., Shashikumar N., Mamatha V., Rose Johnson A. (2023). Polyfunctional CD4 T-cells correlating with neutralising antibody is a hallmark of COVISHIELDTM and COVAXIN^®^ induced immunity in COVID-19 exposed Indians. Npj Vaccines.

[12] Leng A., Shah M., Ahmad S.A., Premraj L., Wildi K., Li Bassi G., Pardo C.A., Choi A., Cho S.-M. (2023). Pathogenesis Underlying Neurological Manifestations of Long COVID Syndrome and Potential Therapeutics. Cells.

[13] Planchuelo-Gómez Á., Trigo J., de Luis-García R., Guerrero Á.L., Porta-Etessam J., García-Azorín D. (2020). Deep Phenotyping of Headache in Hospitalized COVID-19 Patients via Principal Component Analysis. Front. Neurol..

[14] Trigo López J., García-Azorín D., Planchuelo-Gómez Á., García-Iglesias C., Dueñas-Gutiérrez C., Guerrero Á.L. (2020). Phenotypic Characterization of Acute Headache Attributed to SARS-CoV-2: An ICHD-3 Validation Study on 106 Hospitalized Patients. Cephalalgia.

[15] Mutiawati E., Kusuma H.I., Fahriani M., Harapan H., Syahrul S., Musadir N. (2022). Headache in Post-COVID-19 Patients: Its Characteristics and Relationship with the Quality of Life. Medicina.

[16] Caronna E., Van Den Hoek T.C., Bolay H., Garcia-Azorin D., Gago-Veiga A.B., Valeriani M., Takizawa T., Messlinger K., Shapiro R.E., Goadsby P.J. (2023). Headache Attributed to SARS-CoV-2 Infection, Vaccination and the Impact on Primary Headache Disorders of the COVID-19 Pandemic: A Comprehensive Review. Cephalalgia.

[17] García-Azorín D., Sierra Á., Trigo J., Alberdi A., Blanco M., Calcerrada I., Cornejo A., Cubero M., Gil A., García-Iglesias C. (2021). Frequency and Phenotype of Headache in COVID-19: A Study of 2194 Patients. Sci. Rep..

[18] Rodrigues A.N., Dias A.R.N., Paranhos A.C.M., Silva C.C., Bastos T.D.R., Brito B.B.D., Da Silva N.M., De Sousa E.D.J.S., Quaresma J.A.S., Falcão L.F.M. (2023). Headache in Long COVID as Disabling Condition: A Clinical Approach. Front. Neurol..

[19] Fujita K., Otsuka Y., Sunada N., Honda H., Tokumasu K., Nakano Y., Sakurada Y., Obika M., Hagiya H., Otsuka F. (2023). Manifestation of Headache Affecting Quality of Life in Long COVID Patients. JCM.

[20] Sunada N., Nakano Y., Otsuka Y., Tokumasu K., Honda H., Sakurada Y., Matsuda Y., Hasegawa T., Omura D., Ochi K. (2022). Characteristics of Sleep Disturbance in Patients with Long COVID: A Retrospective Observational Study in Japan. JCM.

[21] Nakano Y., Otsuka Y., Honda H., Sunada N., Tokumasu K., Sakurada Y., Matsuda Y., Hasegawa T., Ochi K., Hagiya H. (2022). Transitional Changes in Fatigue-Related Symptoms Due to Long COVID: A Single-Center Retrospective Observational Study in Japan. Medicina.

[22] Aparisi Á., Ybarra-Falcón C., Iglesias-Echeverría C., García-Gómez M., Marcos-Mangas M., Valle-Peñacoba G., Carrasco-Moraleja M., Fernández-de-las-Peñas C., Guerrero Á.L., García-Azorín D. (2022). Cardio-Pulmonary Dysfunction Evaluation in Patients with Persistent Post-COVID-19 Headache. Int. J. Environ. Res. Public Health.

[23] Swank Z., Senussi Y., Manickas-Hill Z., Yu X.G., Li J.Z., Alter G., Walt D.R. (2023). Persistent Circulating Severe Acute Respiratory Syndrome Coronavirus 2 Spike Is Associated With Post-Acute Coronavirus Disease 2019 Sequelae. Clin. Infect. Dis..

[24] Stein S.R., Ramelli S.C., Grazioli A., Chung J.-Y., Singh M., Yinda C.K., Winkler C.W., Sun J., Dickey J.M., Ylaya K. (2022). SARS-CoV-2 Infection and Persistence in the Human Body and Brain at Autopsy. Nature.

[25] Planchuelo-Gómez Á., Garcia-Azorín D., Aja-Fernándes S., Rodríguez M., Guerrero A.L., Moro R., De Luis-Garcia R. White Matter Microstructural Alterations in Patients with Persistent Headache after COVID-19 Infection: An Exploratory Study. International Headache Society & European Headache Federation. Proceedings of the International Headache Congress.

[26] Grundmann A., Wu C., Hardwick M., Baillie J.K., Openshaw P.J.M., Semple M.G., Böhning D., Pett S., Michael B.D., Thomas R.H. (2023). Fewer COVID-19 Neurological Complications with Dexamethasone and Remdesivir. Ann. Neurol..

[27] Al-Hashel J.Y., Ismail I.I. (2020). Impact of Coronavirus Disease 2019 (COVID-19) Pandemic on Patients with Migraine: A Web-Based Survey Study. J. Headache Pain.

[28] Pelayo-González H.J., Reyes-Meza V., Méndez-Balbuena I., Méndez-Díaz O., Trenado C., Ruge D., García-Aguilar G., López-Cortés V.A. (2023). Quantitative Electroencephalographic Analysis in Women with Migraine during the Luteal Phase. Appl. Sci..

